# Smartphones, the Epidemic of the 21st Century: A Possible Source of Addictions and Neuropsychiatric Consequences

**DOI:** 10.3390/ijerph19095152

**Published:** 2022-04-23

**Authors:** Klaudia Adamczewska-Chmiel, Katarzyna Dudzic, Tomasz Chmiela, Agnieszka Gorzkowska

**Affiliations:** 1 Students’ Scientific Association, Department of Neurology, Faculty of Medical Sciences in Katowice, Medical University of Silesia, 40-752 Katowice, Poland; klaudia.adamczewska75@gmail.com; 2 Students’ Scientific Association, Department of Neurorehabilitation, Faculty of Medical Sciences in Katowice, Medical University of Silesia, 40-752 Katowice, Poland; dudzic.kat@gmail.com; 3 Department of Neurology, Faculty of Medical Sciences in Katowice, Medical University of Silesia, 40-752 Katowice, Poland; 4 Department of Neurorehabilitation, Faculty of Medical Sciences in Katowice, Medical University of Silesia, 40-752 Katowice, Poland; agorzkowska@sum.edu.pl

**Keywords:** phonoholism, headache, depression, anxiety, sleep disorders

## Abstract

Background and Objectives: Phonoholism is the excessive and harmful use of a smartphone. We are now observing this phenomenon among adults more often. Using a smartphone for several hours may lead to somatic and psychological symptoms, such as headaches and depression. The aim of this study is to assess the prevalence of phonoholism and to assess the association between smartphone overuse and neuropsychiatric disorders. Materials and Methods: A total of 368 people (70.1% were woman), aged between 19 and 82 years (average age 26.1), took part in an anonymous questionnaire consisting of the following elements: Hospital Anxiety and Depression Scale (HADS), Mobile Phone Problem Use Scale (MPPUS-9), and original questions regarding headaches and sleep quality, along with a subjective assessment of the use of smartphones and an objective evaluation based on data from the applications “Stay Free” and “Screen Time”. Results: A total of 61 respondents (16.6%) obtained a score on the MPPUS-9 scale, which revealed their problematic use of mobile devices. Patients with phonoholism had significantly more headaches (85% vs. 58.7%, *p* = 0.027). Subjects with phonoholism had significantly shorter mean sleep duration (7.14 h vs. 7.42 h, *p* = 0.0475) and were less likely to feel sleepy during the day (43.33% vs. 59.73%, *p* = 0.0271). The group with phonoholism had significantly higher scores on the HADS-A anxiety scale (8.29 vs. 10.9, *p* = 0.015), but a statistical significance was not confirmed for depressive symptoms. Conclusions: The excessive use of the telephone negatively affects both somatic and mental health and can pose a significant clinical problem.

## 1. Introduction

Due to technological development and the spread of portable multimedia devices, smartphones are now universally used. The estimated worldwide number of smartphone users in 2021 was >3.8 billion, a number that has doubled since 2015 [1]. Despite the fact that people benefit from the wide range of applications that are available on smartphones, the growing popularity of smartphones may lead to their overuse. Indeed, problematic smartphone use (PSU), defined as the excessive use of mobile devices with a negative impact on academic, professional, and/or social functioning [2], has become an increasing problem. Despite the prevalence of this phenomenon, there is still no widely accepted definition and terms such as smartphone dependence syndrome and smartphone addiction are not included in current official classifications of diseases, such as ICD-10 or DSM-5 [3]. As early as 1996, this problem was classified as falling within the category of technological addictions and considered as “a behavioural addiction, characterised by the dependence between a person and a device, in the absence of a simultaneous physical intoxication” [4]. On the other hand, other researchers point out that problematic smartphone use is a heterogeneous and multifaceted phenomenon and should be better studied, as little evidence supports its affiliation with behavioural disorders similar to, for instance, drug addiction [2].

Despite the unquestionable advantages of using smartphones, one should remember the examples of its negative influence on people, such as the disruption of interpersonal relations, withdrawal from the outside world due to limited direct contact with other people, and the increased amount of time spent alone compared to time spent in a group. Using a smartphone to participate in social life may lead to the establishment of superficial relationships as well as the loss of skills to create complex statements and appropriate stylistic features of messages and spelling [5]. An additional negative aspect of increased smartphone usage is the exposure to the magnetic radiation emitted by mobile devices [5]. A growing number of studies indicate that smartphone abuse is associated with additional stress, sleep disturbances, lowered mood, and depression [6]. It is noteworthy that during the COVID-19 pandemic, the prevalence of the aforementioned disorders increased [7]. In situations of prolonged stress, one of the ways of coping is to resort to activities that make it possible to reduce negative emotions, which in predisposed individuals may lead to problematic habits [8]. Furthermore, in a study by J. Wang et al., it was shown that the risk of headaches increased by 38% percent in those who used smartphones compared to those who did not [9]. This problem not only affects adults, as a growing number of studies report that this problem is becoming more prevalent among a population of increasingly younger children [10].

The aim of this study is to assess the prevalence of phonoholism among smartphone users and to assess the association between smartphone overuse and neuropsychiatric disorders. In our study, we focused solely on adults. To the best of our knowledge, this is the first study to investigate the problematic use of mobile devices on an adult population in Poland. Furthermore, this work not only studies the occurrence of phonoholism and its neuropsychiatric consequences, but also confronts these issues with the use of smartphones through the real and objective measurement of mobile device usage time.

## 2. Materials and Methods

We employed a cross-sectional study to assess association between smartphone usage and neuropsychiatric disorders—headaches, sleep disorders, mood and anxiety disorders. The participants included adult smartphone users in Poland. The data were collected from 24 February 2021 to 1 October 2021 through a self-administered questionnaire. A voluntary response sampling method was used to recruit participants. Volunteers were recruited online through social media and the participants were also encouraged to share the questionnaire further. Smartphone users aged over 18 years were included in this study. Exclusion criteria were as follows: diagnosed depression, anxiety, or sleep disorders.

The study involved a questionnaire containing two validated clinical scales: the Hospital Anxiety and Depression Scale (HADS), a brief scale evaluating anxiety and depression, and the Mobile Phone Problem Use Scale (MPPUS-9), the Polish version of MPPUS-10, which has been validated and adapted to Polish conditions. This study also contained a set of original questions regarding headaches, sleep quality, and the subjective assessment of smartphone use. A Numeric Pain Rating Scale (NRS) was used to assess the severity of the headaches. The respondents answered the questions online. All questions were closed-ended, which means that the respondents were only allowed to select answers from a strictly defined set of options.

Respondents were asked to use the following free applications that they had installed on their smartphones: “Stay Free” for Android users and “Screen Time” for iOS users. These applications provide information on the total number of hours spent in front of a screen over the previous seven days, the daily average over the previous week, and the most frequently used apps. The provided answers served only to collect statistical data. Participants provided informed consent electronically.

A total of 397 people responded to the questionnaire, and 29 individuals were excluded from the study (17 were excluded due to incorrect survey completion and 12 were excluded due to inappropriate age (<18 years old)). The final study group consisted of 368 people, including 251 females (70.1%) and 107 males (29.9%) aged between 19 and 82 (mean age 26.1) years. For statistical calculations, we divided the subjects into two groups based on their MPPUS-9 scale scores: <53 points and ≥53 points. The cut-off point of ≥53 indicated the presence of problematic smartphone use [11].

The statistical analysis was performed with Statistica 13.3 (TIBCO Software Inc., Palo Alto, CA, USA, (2017) (data analysis software system, version 13. http://statistica.io)). The quantitative variables are presented as an arithmetic mean and a standard deviation. The qualitative variables are presented as absolute values and percentages. The normality of distribution was assessed with the Shapiro–Wilk test.

Due to the fact that the normal distribution in the analysed groups was not confirmed, the intergroup differences for the quantitative variable were assessed with the Mann–Whitney Utest. Fisher’s exact test and the chi-square test was performed to assess the qualitative variables.

Due to the survey character of the work and data anonymisation, the Ethics Committee of the Medical University of Silesia waived the requirement to obtain an ethical approval for this study.

## 3. Results

### 3.1. Prevalence of Phonoholism

A total of 61 individuals (16.6%), of which 47 were women and 16 were men, met the criteria for a diagnosis of phonoholism defined as an MPPUS-9 score of over 53 points. There were no statistically significant differences between the two groups regarding gender, education, physical activity, comorbidities, and place of residence. Detailed data are summarised in Table 1.

### 3.2. Phonoholism and Headache

Among the patients with phonoholism, a significantly higher number of individuals had headaches (85% vs. 58.7%, *p* = 0.027). However, there was no statistically significant difference in the frequency of pain among patients with pain in these two groups. In the group of patients without phonoholism, nausea (22.9% vs. 7.8%, *p* = 0.33), photosensitivity (34.9% vs. 15.7%, *p* = 0.29), and irritability (38.9% vs. 15.7%, *p* = 0.12) often accompanied headaches and fatigue, which was close to statistical significance (42.9% vs. 23.5%, *p* = 0.050). There was no difference in pain localisation. Patients dependent on mobile devices use often described pain as throbbing (56.0% vs. 25.5% *p* = 0.006) or taking the form of tension (12.6% vs. 1.96% *p* = 0.037). Detailed data are summarised in Table 2.

### 3.3. Phonoholism and Sleep Disturbances

Individuals with phonoholism had significantly shorter mean sleep duration (7.14 h vs. 7.42 h, *p* = 0.0475) and were less likely to feel well rested during the day (43.33% vs. 59.73%, *p* = 0.0271). There was no statistically significant difference in the prevalence of daytime sleepiness, trouble falling asleep, or mean time of falling asleep. Detailed data are summarised in Table 3.

### 3.4. Mood and Anxiety Disorders Related to Phonoholism

The group with phonoholism achieved substantially higher scores on the HADS-A anxiety scale (8.29 vs. 10.9 points, *p* = 0.015). A statistical significance was not proven for the component ‘depressive symptoms’ of the HADS-D (6.17 vs. 8.05 points, *p* = 0.1589). Detailed data are summarised in Table 4.

### 3.5. Phonoholism and Mobile Device Usage

In the group with phonoholism, subjects declared a significantly longer time of mobile device use, which was consistent with the objective measurements of the applications installed in the subjects’ devices (3 h 57 min vs. 4 h 38 min, *p* = 0.0497).

People with phonoholism were more likely to watch movies on mobile devices (33.9% vs. 55.0%), with no differences for other activities. Among the most frequently used apps (data collected from applications installed in the subjects’ devices), shopping applications were more common among addicts (2.0% vs. 5.2%, *p* = 0.012), but no significant differences were found regarding other apps. No differences were observed concerning the time of day of phone usage. Detailed data are summarised in Table 5.

## 4. Discussion

In this study, we investigated the possible negative effect of excessive smartphone use on somatic and mental health. Regarding neurological disorders, the results of our study support the fact that excessive smartphone use increases the risk of headaches. Similar findings were presented in meta-analysis that concluded that smartphone users had an increased risk of headaches compared to non-users. Among smartphone users, the risk of headaches was also greater in those who had a longer duration of calls per day and a higher frequency of calls per day [9]. In another study, it was shown that headaches were more common in people who used smartphones frequently [12]. In addition, the duration and frequency of headache attacks were higher in people who frequently used mobile devices and they used analgesics more often to relieve their headaches [12]. In our study, we found that headaches were more frequent in the group of phonoholics, but there was no difference in their intensity. Interestingly, differences in the nature of pain were observed in this study. It was more often described as throbbing or tension in the group of non-phonoholics, and symptoms such as photosensitivity, irritability and nausea were experienced less often by phonoholics. Another study showed that headaches related to smartphone overuse showed mostly stereotyped clinical features, such as mild intensity, a dull or pressing quality, and ipsilateral location [13]. We also performed an analysis that revealed phonoholics were more likely to watch videos on smartphones than non-phonoholics, which may further influence the fact that phonoholics use smartphones for longer durations. According to current research, factors such as radiofrequency (RF) fields, noise, psychological factors or temperature changes could induce headaches related to the use of smartphones [12]. The effect of radiofrequency on the occurrence of headaches is debatable. A meta-analysis of 17 studies containing 1174 subjects found no effect of short-term exposure to RF radiation on the incidence of headaches [13]. The COSMO prospective cohort study of 24,169 subjects did not confirm the effect of long-term RF exposure on the occurrence of headaches [14]. Some studies have confirmed the effect of RF on the increased risk of headaches. Potential mechanisms by which FR could affect the incidence of pain include decreased regional cerebral cellular blood flow with increased blood flow in the prefrontal cortex, altered brain oscillatory responses, and the disruption of the energetic brain metabolism [15]. A study of 25,751 workers exposed to prolonged noise at work confirmed the strong influence of this factor on the occurrence of headaches. Noise is considered to disrupt the neurovascular system and induce abnormal muscle strain [16]. Noise can also cause headaches through its effect on the human psyche. It has indirect signalling pathways to the limbic cortex, autonomic nervous system and neuroendocrine system that are closely linked to stress response. Chronic exposure to stress may induce central sensitisation, the depletion of the pain control system, and hyperalgesia, which could contribute to more frequent headache recurrence [16,17,18].

Smartphone use may also change head posture and neck mobility, which may lead to headaches [13]. Furthermore, other headache triggers, such as anxiety, depression and sleep disturbance, should be considered.

Studies have shown that phonoholics are also more likely to experience symptoms typical of depression and anxiety disorders. A study that examined student populations found that anxiety symptoms were positively correlated with problematic smartphone use [19]. Furthermore, the authors in the latter paper demonstrated that self-efficacy might mediate the relationship between anxiety disorders and PSU [20]. Nevertheless, the modulating effect of self-efficacy on the relationship between anxiety symptoms and PSU was insignificant [19]. A systematic review revealed a correlation between smartphone use and stress and anxiety and concluded that the severity of both depression and anxiety were substantially associated with excessive smartphone usage [20,21,22]. In our study, we confirmed the association of phonoholism and anxiety, but we did not obtain a statistically significant difference in the case of mood disorders; however, it should be noted that we used other clinical scales to assess these disorders. In another study, individuals that displayed excessive smartphone usage scored higher on the Beck Depression Index (BDI), the Beck Anxiety Index (BAI) scale, and the daytime dysfunction component of the Pittsburgh Sleep Quality Index (PSQI) scale in comparison with individuals in the group that used smartphones to a lesser extent [23]. The results of this study showed that excessive smartphone use can lead to depression and/or anxiety, both of which are linked to sleep problems [23].

The results of our study likewise indicate that phonoholics tended to suffer from more frequent sleep disturbance. Another study found an association between smartphone use for gaming, surfing, and texting in bed and increased insomnia symptoms [24], possibly indicating a delayed sleep phase [25]. In our study, phonoholics had a shorter mean sleep duration and experienced sleep deprivation more often than other participants. It has been hypothesised that this could be due to the magnetic field emitted by smartphones, which could negatively affect serum melatonin levels (an important factor for sleep) and cerebral blood flow, affecting the quality of sleep of phonoholics [19,26]. Addicts may remain in a constant state of anticipation of incoming phone messages, which can also result in the desynchronisation of sleep rhythms [27,28]. Furthermore, social media interactions can trigger excitement and induce mood changes, all of which have the potential to disrupt sleep [27,28,29]. However, our study did not corroborate a connection between this specific activity and phonoholism and the occurrence of sleep disorders, but it was correlated with total screen time.

Among the most commonly used applications, the use of shopping apps was more prevalent in addicts than in non-addicts. Compulsive buying can be conceptualised as an addiction because it contains the same elements that behavioural addiction possesses [30]. It was observed that shopping enhanced people’s mood, which, in turn, improved their self-esteem. The elevated state experienced during shopping can be viewed as a critical motivating element for this addiction. These findings indicate that debt and financial instability were evident negative consequences of such behaviour [30]. It is worth adding that shopping addictions develop gradually, when the shopper occasionally purchases and spends money in an attempt to escape from unpleasant emotions or boredom [31,32]. Smartphone addiction develops as a long-term process similar to behavioural addiction [32,33]. Dependency often begins with a seemingly benign behaviour (shopping, Internet and/or smartphone use) that, through a variety of psychological, biophysical, and/or environmental triggers, can become harmful and evolve into an addiction [33,34,35].

The present study has some limitations that should be considered. First, this study relied on a convenience sample and the data were collected online, leading to the possibility of self-selection bias. Secondly, all participants were adults and most were young and well educated, with a large group of university students, which may prevent the generalisation of the results to the public. Longitudinal studies and multi-samples of different educational and age backgrounds are needed. The MPPUS-9 scale used in this study was recently validated for the Polish population version of the MPPUS-10 scale. This is a rapid screening tool to assess problematic use of smartphones and has shown a significant correlation with other scales used in the Polish population: MPAAQ (Mobile Phone Addiction Assessment Questionnaire) and IAT (Internet Addiction Test) [11]. However, due to the lack of a universal definition of phonoholism and differences in diagnostic criteria, there may be inconsistencies between results of different scales design to measure this phenomenon [11]. It is worth noting that the MPPUS scale was designed when smartphones were not available and smartphones have many functions that go beyond talking and writing short text messages. Some researchers have shown that smartphone addiction and Internet addiction overlap [36,37]. It is worth emphasizing that the Polish version of the MPPUS-9 scale should be considered a screening rather than a diagnostic tool and obtaining a score above the proposed threshold should be followed by further, more detailed clinical assessment. The MPPUS scale does not identify other psychopathological problems such as personality disturbances or stress levels; therefore, some of the data obtained may be a consequence of these variables rather than directly reflecting the excessive use of mobile phones [11]. It should be emphasized that the selected psychometric instruments may be inadequate to catch the full extent of neuropsychiatric and cognitive consequences of phonoholism. The full assessment of the phenomenon should be extended to include scales that take into account the aforementioned disorders, e.g., Millon Clinical Multiaxial Inventory (MCMI) [38]. Further research taking these aspects into account is needed in order to fully assess the phenomenon of phonoholism. Furthermore, studies involving brain and mind imaging and measurement performed premorbidly and post severe phonoholism are advisable.

## 5. Conclusions

This study demonstrated that phonoholism is a potential risk factor for somatic and mental health deterioration in addicted individuals. Such a negative impact should draw the attention of the larger population and patients with problems that could potentially arise from the overuse of smartphones.

## Figures and Tables

**Table 1:** Group comparison between individuals that meet the criteria for phonoholism (MPPUS-9 ≥ 53) and a group of participants that does not meet the criteria for phonoholism (MPPUS-9 < 53), regarding demographics. Mann–Whitney U test was performed for quantitative variables and Fisher’s exact test was performed for qualitative variables.

Variable	MPPUS-9 < 53 pt.	MPPUS-9 ≥ 53 pt.	*p*-Value
Age (years)	26.5 ± 8.6	24.1 ± 3.2	0.643
Gender *n* (%) Woman Man			
	206 (69.1)	45 (73.8)	0.466
	92 (30.9)	16 (26.2)	
Education *n* (%) Primary			
	2 (0.7)	0 (0.0)	0.425
Vocational Secondary Student Higher	1 (0.3)	1 (1.6)	
	25 (8.4)	3 (4.9)	
	205 (68.8)	46 (75.4)	
	66 (22.2)	10 (16.4)	
Residence *n* (%)			
Rural	64 (21.5)	6 (9.8)	0.402
City up to 50 thous.	69 (23.2)	18 (29.5)	
City up to 100 thous.	23 (7.7)	6 (9.840	
City up to 250 thous.	56 (18.8)	11 (18.0)	
City over 250 thous.	87 (29.2)	19 (24.6)	
Comorbid chronic diseases	61 (20.5)	15 (24.6)	0.532
Physical activity *n* (%)			
>1 h	49 (16.4)	12 (19.7)	0.320
1–2 h	48 (16.1)	16 (26.3)	
2–3 h	60 (20.1)	12 (19.7)	
3–4 h	49 (16.4)	9 (14.8)	
4–5 h	38 (12.8)	6 (9.8)	
5–6 h	29 (9.7)	2 (3.3)	
<6 h	26 (8.7)	3 (4.9)	

**Table 2:** Group comparison between individuals that meet the criteria for phonoholism (MPPUS-9 ≥ 53) and a group of participants that does not meet the criteria for phonoholism (MPPUS-9 < 53) regarding the occurrence and characteristics of headaches. Mann–Whitney U tests were performed for quantitative variables, and Fisher’s exact tests were performed for qualitative variables.

Variable	MPPUS-9 < 53 pt.	MPPUS-9 ≥ 53 pt.	*p*-Value
Presence of headaches *n* (%)	175 (58.7)	51 (83.6)	0.027
Frequency of headaches *n* (%)			
1 x/month	31 (17.7)	9 (17.70)	0.478
2–3 x/month	76 (43.4)	18 (35.3)	
4–6 x/month	37 (21.1)	16 (31.3)	
<6 x/month	31 (17.7)	8 (15.7)	
Severity of headaches (1–10) *n* (%)	5.06 ± 2.1	5.33 ± 1.9	0.286
Symptoms accompanying headache	98 (56.0)	32 (62.7)	0.576
Nausea	40 (22.9)	4 (7.8)	0.033
Vomiting	5 (2.9)	0 (0.0)	0.227
Photosensitivity	61 (34.9)	8 (15.7)	0.029
Sound hypersensitivity	43 (24.6)	7 (13.7)	0.147
Irritability	68 (38.9)	8 (15.7)	0.012
Visual disturbances	22 (12.6)	2 (3.9)	0.095
Whirling sensation	14 (8.0)	3 (5.9)	0.628
Fatigue	75 (42.9)	12 (23.5)	0.050
Pain localisation *n* (%)			
Frontal region	131 (74.9)	45 (73.8)	0.341
Ocular region	58 (33.1)	14 (22.95)	0.526
Parietal region	59 (33.7)	15 (24.6)	0.624
Occipital region	30 (17.1)	12 (19.6)	0.352
Mandibular region	4 (2.3)	2 (3.3)	0.528
Character of pain *n* (%)			
Stabbing	13 (7.4)	3 (5.9)	0.715
Compression	99 (56.6)	21 (41.2)	0.184
Crushing	39 (22.3)	5 (9.8)	0.076
Thunderclap	44 (25.1)	10 (19.6)	0.477
Throbbing	98 (56.0)	13 (25.5)	0.006
Tension	22 (12.6)	1 (1.96)	0.037

**Table 3:** Group comparison between individuals that meet the criteria for phonoholism (MPPUS-9 ≥ 53) and a group of participants that does not meet the criteria for phonoholism (MPPUS-9 < 53) regarding sleep disorders. Mann–Whitney U test was performed for quantitative variables and Fisher’s exact test was performed for qualitative variables.

Variable	MPPUS-9 < 53 pt.	MPPUS-9 ≥ 53 pt.	*p*-Value
Mean sleep duration (h)	7.43 ± 1.2	7.15 ± 1.5	0.048
Mean time of falling asleep (min)	24.7 ± 28.5	24.2 ± 19.0	0.152
Feeling well rested *n* (%)	178 (59.7)	23 (37.7)	0.035
Daytime sleepiness *n* (%)	170 (57.1)	46 (75.4)	0.095
Problems falling asleep *n* (%)	143 (48.0)	36 (59.0)	0.274

**Table 4:** Group comparison between individuals that meet the criteria for phonoholism (MPPUS-9 ≥ 53) and a group of participants that does not meet the criteria for phonoholism (MPPUS-9 < 53) regarding HADS scale results. Mann–Whitney U test was performed for quantitative variables and Fisher’s exact test was performed for qualitative variables.

	MPPUS-9 < 53 pt.	MPPUS-9 ≥ 53 pt.	*p* Value
HADS-A	8.3 ± 4.6	10.9 ± 4.5	0.015
HADS-D	6.2 ± 4.1	8.1 ± 4.4	0.159

**Table 5:** Group comparison between individuals that meet the criteria for phonoholism (MPPUS-9 ≥ 53) and a group of participants that does not meet the criteria for phonoholism (MPPUS-9 < 53) regarding the pattern of smartphone usage. Mann–Whitney U test was performed for quantitative variables and Fisher’s exact test was performed for qualitative variables.

Variable	MPPUS-9 < 53 pt.	MPPUS-9 ≥ 53 pt.	*p*-Value
**Duration of usage (declared) [n (%)]**			
>1 h	19 (6.4)	0 (0.0)	0.006
1–2 h	36 (12.1)	2 (3.3)	
2–3 h	63 (21.1)	6 (9.8)	
3–4 h	53 (17.8)	13 (21.3)	
4–5 h	47 (15.8)	17 (27.9)	
5–6 h	42 (14.1)	9 (14.8)	
<6 h	38 (12.8)	13 (21.3)	
**Duration of usage (measurement) (h)**	3 h 57 min ± 2 h 19 min	4 h 38 min ± 2 h 1 min	0.0497
**Purpose of mobile device usage (n (%))**			
Calls	162 (54.4)	26 (42.6)	0.242
SMS/MMS	136 (45.6)	23 (37.7)	0.389
Browser use	227 (76.1)	46 (75.4)	0.934
Social media	263 (88.3)	59 (96.7)	0.540
Work	43 (14.4)	10 (16.4)	0.723
Videos	101 (33.9)	33 (54.1)	0.020
Education	119 (39.9)	24 (39.3)	0.935
Music	194 (65.1)	36 (59.0)	0.576
Photography/filming	76 (25.5)	19 (31.2)	0.443
Games	76 (25.5)	19 (31.2)	0.443
**Most used applications (n (%))**			
Communicators	153 (51.3)	24 (39.3)	0.218
Social media	196 (65.8)	33 (54.1)	0.290
Games	31 (10.4)	7 (11.5)	0.821
Browsers	83 (27.9)	16 (26.3)	0.816
Streaming media	91 (30.5)	16 (26.3)	0.566
Shopping	6 (2.0)	5 (8.2)	0.012
Navigation	12 (4.0)	2 (3.3)	0.784
Mail	7 (2.4)	1 (1.64)	0.733
Banking	4 (1.3)	1 (1.64)	0.860
Photos	4 (1.3)	0 (0.0)	0.365
Education	6 (2.0)	3 (4.9)	0.194

## Data Availability

The data presented in this study are available on request from the corresponding author.
